# Supplementary material for: An optimised faecal microRNA sequencing pipeline reveals fibrosis in Trichuris muris infection
Source: Nat Commun. 2025 Feb 12;16:1589. doi: 10.1038/s41467-025-56698-w (PMC11822213; doi:10.1038/s41467-025-56698-w)
Supplement: Supplementary file 1 — Supplementary Information [file 41467_2025_56698_MOESM1_ESM.pdf]

An Optimised Faecal microRNA Sequencing Pipeline Reveals Fibrosis in *Trichuris muris* Infection

Supplemental information

Target	Metal Isotope	Company	Catalogue Number	Concentration	Dilution Factor
αSMA	141Pr	Standard Biotools	3141017D	0.500mg/mL	150
Collagen VI	142Nd	Abcam	ab229450	0.433mg/mL	50
Heparan Sulphate	143Nd	Amsbio	270255-1	0.352mg/mL	50
Collagen I	169Tm	Standard Biotools	3169023D	0.500mg/mL	200
Biotinylated Hyaluronan-Binding Protein (HABP)	N/A	Merck	385911	0.500mg/mL	100
Anti-Biotin	170Er	Biolegend	409002	0.737mg/mL	100

Table 1. *Antibody concentrations and dilutions used for Hyperion® mass cytometry.*

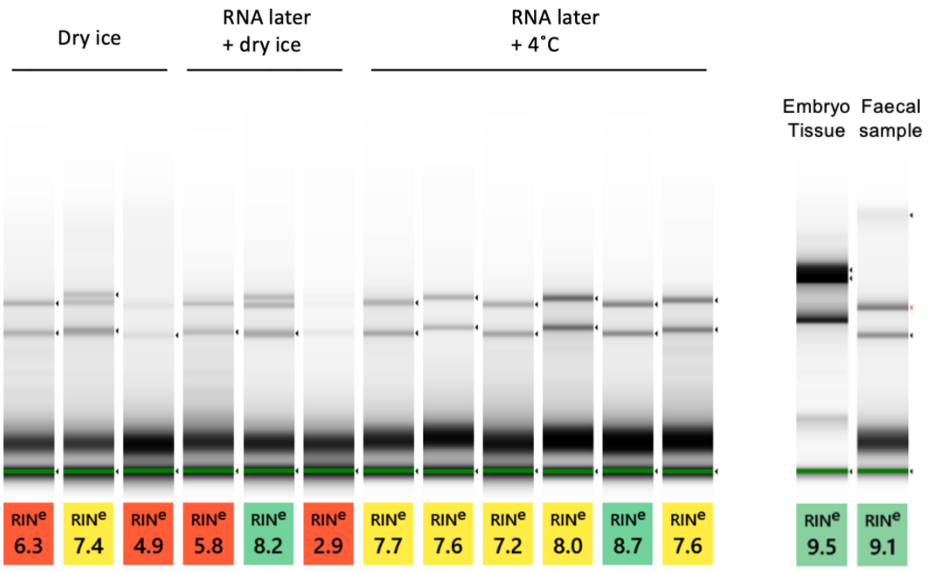

Figure 1. *Electropherogram of Faecal Total RNA Extracted After Different Storage Conditions.* The TapeStation gel electropherogram shows two RNA bands for the large (upper band) and small (lower band) ribosomal subunits. The thicker, darker and more diffuse bands at the bottom of the electropherogram represents the small RNA content of each sample. (RNA INtegrity) RINe values are calculated by the TapeStation from 1 to 10 with 10 being the highest quality RNA. All total RNA samples in the left panel are from murine faecal samples and in the right panel the RNA content of a faecal sample is compared to that of murine embryo tissue.

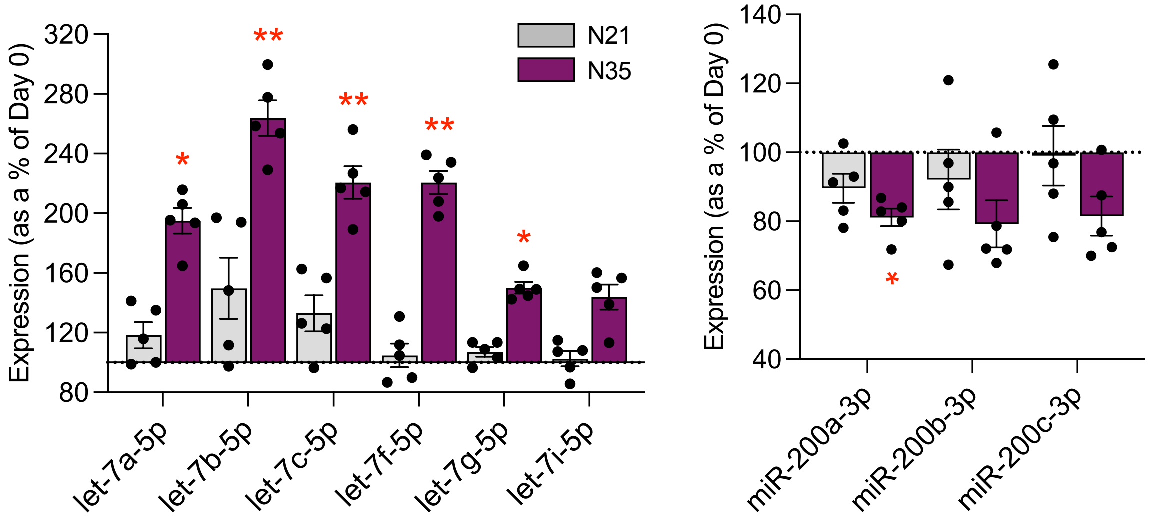

Figure 2. *Dynamics of Most Abundant Faecal miRNAs in Healthy Mice Over Time*. Expression of miRNAs on day 21 and day 35 (N21 and N35) shown as a percentage change from the value at day 0 (I0). Only reads that mapped uniquely to each miRNA are included. Mean and SEM are plotted. Statistical significance was calculated on the normalised counts for each group by two-way ANOVA with Geisser-greenhouse correction and Tukey's multiple comparisons test. Significance values displayed are for each timepoint versus I0. * = adj. *p* <0.05, ** = adj. *p* <0.01.

```
Most common miR-6239 read:  agcggtggatcactcggt
                            ||||*|||||||||||||
    6239 genomic locus:    tagcgttggatcactcggtg

Most common miR-6239 read:  agcggtggatcactcggt
                            |||||||||||||||||*
   Mouse 5.8S rRNA locus:  tagcggtggatcactcggct
```

Figure 3. *Alignment of miR-6239 reads to the miR-6239 locus and the mouse rRNA locus***.** Solid lines illustrate matching nucleotides between the read and reference sequence and asterisks represent mismatches.

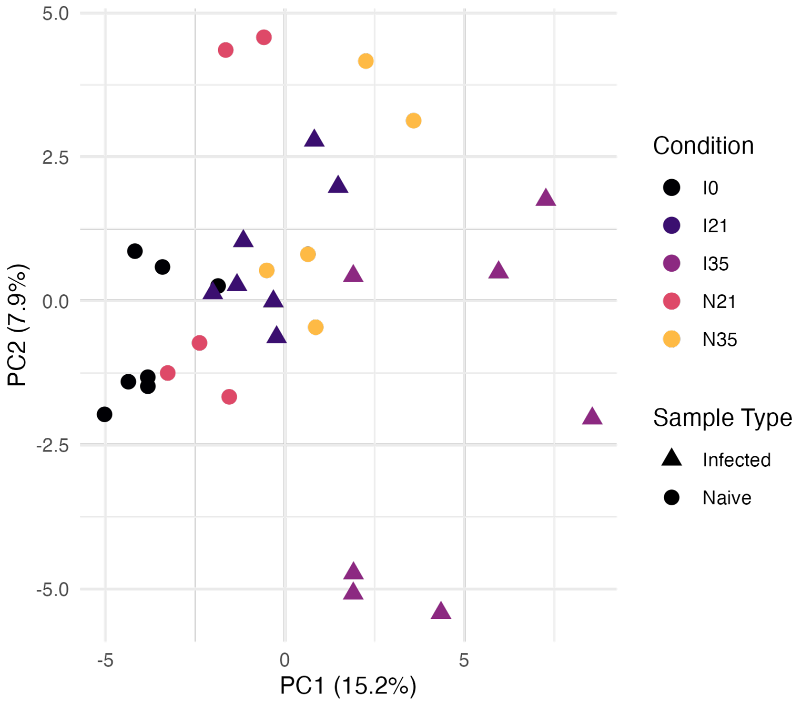

Figure 4. *Principle component analysis of faecal miRNA samples from T. muris-infected and naïve mice.* Samples were taken from three timepoints Day 0 (I0), Day 21 (I21 and N21) and Day 35 (I35 and N35) post-gavage. Data was subject to variance stabilising transformation (VST) prior to plotting.

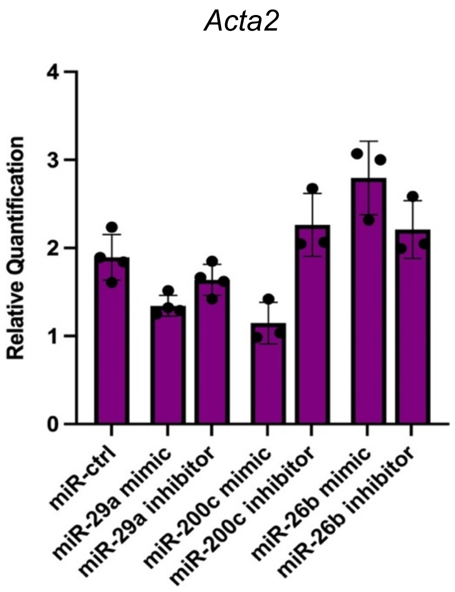

Figure 5. *Transfection of Differentially Expressed miRNAs on Acta2 Expression.* 3T3 fibroblasts were cultured in TGF-β in the presence of 50nM of negative control miRNA (miR-ctrl), miR-29a mimic or inhibitor, miR-200c mimic or inhibitor, or miR-26b mimic or inhibitor for 36 hours. RNA was harvested and the expression of *Acta2* quantified by qPCR relative to the expression in the untreated untransfected cells in each biological replicate. Mean and SD are plotted. Statistical significance was calculated with one-way ANOVA with Geisser-greenhouse correction and Dunnet's multiple comparisons test.

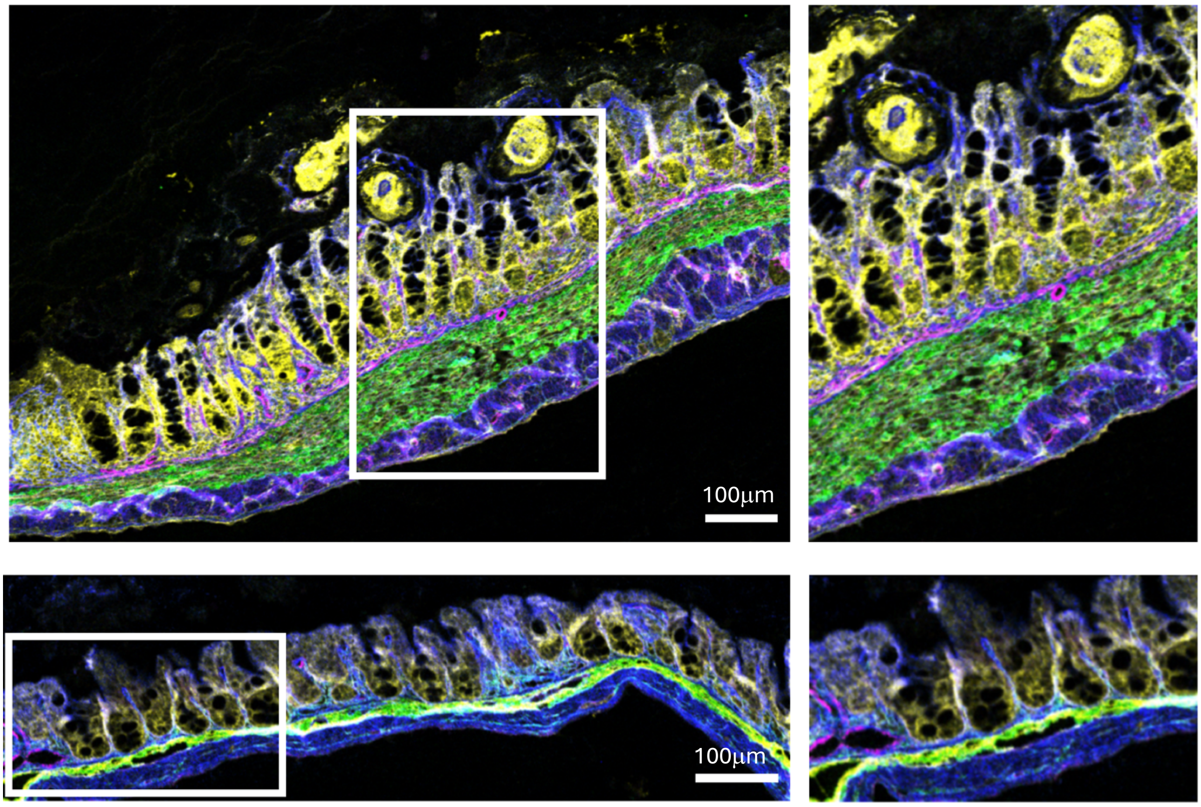

Figure 6. *Zoomed Hyperion® mass cytometry images from Figure 4.*

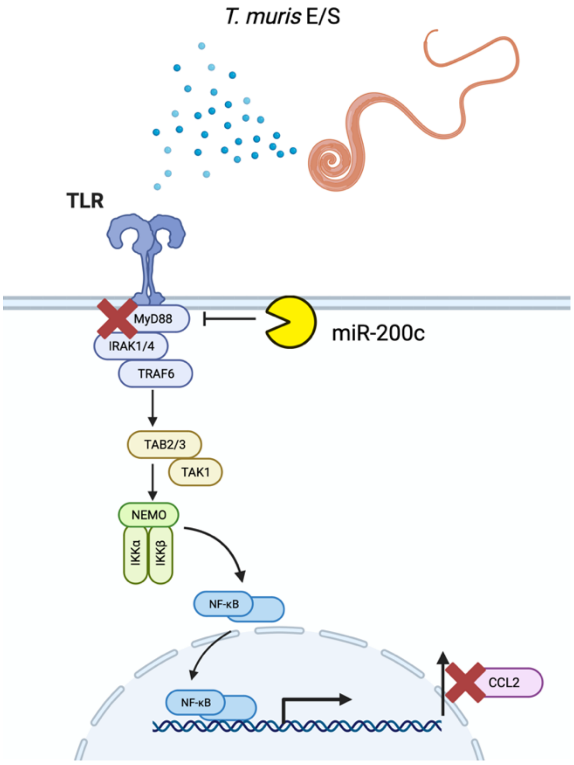

Figure 7. *miR-200c can Prevent T. muris E/S-induced Upregulation of CCL2 by Targeting MyD88. T. muris* E/S activates TLR-MyD88-NF-kB signalling. MyD88 is a known target of miR-200c and its degradation could lead to a decrease in CCL2 production via prevention of NF-kB activation. Created in BioRender. Layton, E. (2024) https://BioRender.com/p84x755

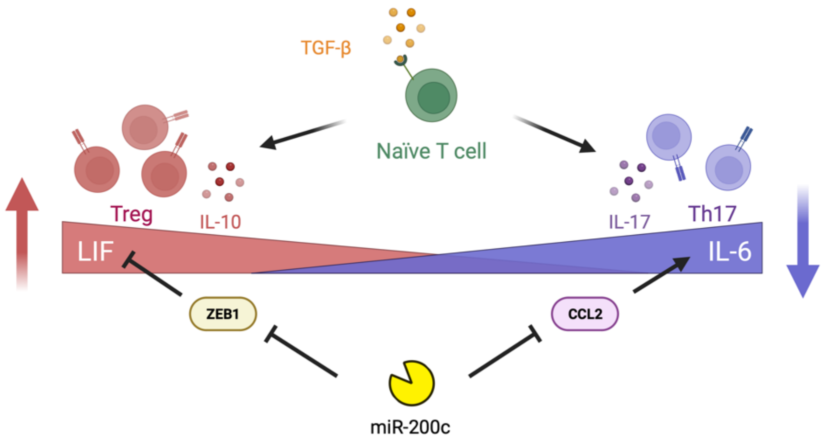

Figure 8. *miR-200c Induces Tregs by Increasing LIF and decreasing IL-6.* In the presence of TGF-β naïve T cells can be differentiated into Tregs or Th17 cells dependent on the level of LIF and IL-6, which have been reported to be mutually exclusive. LIF secretion is upregulated by miR-200c mimic in fibroblasts which is known to target ZEB1, a repressor of LIF, and CCL2, an inducer of IL-6. Therefore miR-200c could increase the production of Tregs from naïve T cells in this model. Created in BioRender. Layton, E. (2024) https://BioRender.com/y85t501
