# Supplementary material for: An optimised faecal microRNA sequencing pipeline reveals fibrosis in Trichuris muris infection
Source: Nat Commun. 2025 Feb 12;16:1589. doi: 10.1038/s41467-025-56698-w (PMC11822213; doi:10.1038/s41467-025-56698-w)
Supplement: Supplementary file 2 — Description of Additional Supplementary Files [file 41467_2025_56698_MOESM2_ESM.pdf]

Description of Additional Supplementary Files

Supplementary Data 1: A file containing faecal microRNA counts and the results of the differential expression analysis for the samples in the current study.

Supplementary Data 2: The mRNA targets of the faecal miRNAs differentially expressed on day 35 of chronic T. muris infection that constitute the enriched pathways illustrated in figure 3b.
