# Supplementary material for: An optimised faecal microRNA sequencing pipeline reveals fibrosis in Trichuris muris infection
Source: Nat Commun. 2025 Feb 12;16:1589. doi: 10.1038/s41467-025-56698-w (PMC11822213; doi:10.1038/s41467-025-56698-w)
Supplement: Supplementary file 5 — Reporting Summary [file 41467_2025_56698_MOESM5_ESM.pdf]

# nature portfolio

Corresponding author(s): Ian Roberts, Anna-Marie Fairhurst and Richard Grencis

Last updated by author(s): Jan 9, 2025

# Reporting Summary

Nature Portfolio wishes to improve the reproducibility of the work that we publish. This form provides structure for consistency and transparency in reporting. For further information on Nature Portfolio policies, see our Editorial Policies and the Editorial Policy Checklist.

## Statistics

For all statistical analyses, confirm that the following items are present in the figure legend, table legend, main text, or Methods section.

| n/a | Confirmed | |
|---|---|---|
| ☐ | ☒ | The exact sample size ($n$) for each experimental group/condition, given as a discrete number and unit of measurement |
| ☒ | ☐ | A statement on whether measurements were taken from distinct samples or whether the same sample was measured repeatedly |
| ☐ | ☒ | The statistical test(s) used AND whether they are one- or two-sided<br>*Only common tests should be described solely by name; describe more complex techniques in the Methods section.* |
| ☒ | ☐ | A description of all covariates tested |
| ☐ | ☒ | A description of any assumptions or corrections, such as tests of normality and adjustment for multiple comparisons |
| ☐ | ☒ | A full description of the statistical parameters including central tendency (e.g. means) or other basic estimates (e.g. regression coefficient) AND variation (e.g. standard deviation) or associated estimates of uncertainty (e.g. confidence intervals) |
| ☐ | ☒ | For null hypothesis testing, the test statistic (e.g. $F$, $t$, $r$) with confidence intervals, effect sizes, degrees of freedom and $P$ value noted<br>*Give P values as exact values whenever suitable.* |
| ☒ | ☐ | For Bayesian analysis, information on the choice of priors and Markov chain Monte Carlo settings |
| ☒ | ☐ | For hierarchical and complex designs, identification of the appropriate level for tests and full reporting of outcomes |
| ☒ | ☐ | Estimates of effect sizes (e.g. Cohen's $d$, Pearson's $r$), indicating how they were calculated |

*Our web collection on statistics for biologists contains articles on many of the points above.*

## Software and code

Policy information about availability of computer code

| | |
|---|---|
| Data collection | Software was not used for data collection. |
| Data analysis | All details of software and the versions used can be found on the github repository referred to in the manuscript https://github.com/elayton13/faecalmiRseq |

For manuscripts utilizing custom algorithms or software that are central to the research but not yet described in published literature, software must be made available to editors and reviewers. We strongly encourage code deposition in a community repository (e.g. GitHub). See the Nature Portfolio guidelines for submitting code & software for further information.

## Data

Policy information about availability of data

All manuscripts must include a data availability statement. This statement should provide the following information, where applicable:

- Accession codes, unique identifiers, or web links for publicly available datasets
- A description of any restrictions on data availability
- For clinical datasets or third party data, please ensure that the statement adheres to our policy

Sequencing data generated during the course of this study is deposited under the Gene Expression Omnibus accession number: GSE267555 and is publicly available.

## Research involving human participants, their data, or biological material

Policy information about studies with human participants or human data. See also policy information about sex, gender (identity/presentation), and sexual orientation and race, ethnicity and racism.

| | |
|---|---|
| Reporting on sex and gender | N/A |
| Reporting on race, ethnicity, or other socially relevant groupings | N/A |
| Population characteristics | N/A |
| Recruitment | N/A |
| Ethics oversight | N/A |

Note that full information on the approval of the study protocol must also be provided in the manuscript.

# Field-specific reporting

Please select the one below that is the best fit for your research. If you are not sure, read the appropriate sections before making your selection.

☒ Life sciences ☐ Behavioural & social sciences ☐ Ecological, evolutionary & environmental sciences

For a reference copy of the document with all sections, see nature.com/documents/nr-reporting-summary-flat.pdf

# Life sciences study design

All studies must disclose on these points even when the disclosure is negative.

| | |
|---|---|
| Sample size | Sample sizes were based on previously published data. Please see:<br>Liu, S., R.M. Rezende, T.G. Moreira, S.K. Tankou, L.M. Cox, M. Wu, A. Song, F.H. Dhang, Z. Wei, G. Costamagna, and H.L. Weiner, Oral Administration of miR-30d from Feces of MS Patients Suppresses MS-like Symptoms in Mice by Expanding Akkermansia muciniphila. Cell Host Microbe, 2019. 26(6): p. 779-794 e8. |
| Data exclusions | No data was excluded |
| Replication | To ensure reproducibility a minimum of three independent replicates were performed for the qPCR data and luminex cytokine analysis. For in vivo work there were biological replicates in the infected and naive groups of mice (n=7 and n=5, respectively). |
| Randomization | Allocation of mice to infection or control groups was random. |
| Blinding | Blinding was not performed since a single researcher (EL) was primarily responsible for data collection and analysis. |

# Reporting for specific materials, systems and methods

We require information from authors about some types of materials, experimental systems and methods used in many studies. Here, indicate whether each material, system or method listed is relevant to your study. If you are not sure if a list item applies to your research, read the appropriate section before selecting a response.

### Materials & experimental systems

| n/a | Involved in the study | |
|---|---|---|
| ☐ | ☒ | Antibodies |
| ☐ | ☒ | Eukaryotic cell lines |
| ☒ | ☐ | Palaeontology and archaeology |
| ☐ | ☒ | Animals and other organisms |
| ☒ | ☐ | Clinical data |
| ☒ | ☐ | Dual use research of concern |
| ☒ | ☐ | Plants |

### Methods

| n/a | Involved in the study | |
|---|---|---|
| ☒ | ☐ | ChIP-seq |
| ☒ | ☐ | Flow cytometry |
| ☒ | ☐ | MRI-based neuroimaging |

## Antibodies

| | |
|---|---|
| Antibodies used | For Hyperion imgaing: Collagen VI (Abcam, catalogue ID: ab229450, clone EPR17072), Heparan Sulphate (AmsBio, catalogue ID: |

| | |
|---|---|
| Antibodies used | 270255-1, clone F58-10E4), Collagen I (Standard Biotools, catalogue ID: 3169023D), alpha-SMA (Standard Biotools, catalogue ID 3141017D) Hyaluronic acid binding protein-biotinylated (Merck, catalogue ID: 385911) and metal-conjugated anti-biotin antibody (Standard Biotools, catalogue ID: 3170003B). For confocal imaging: collagen I (Abcam, catalogue ID: ab270993, clone EPR24331-53). |
| Validation | Each antibody was validated for the application by the manufacturer and details of their use in previous publications can be found on the respective manufacturer websites. |

## Eukaryotic cell lines

Policy information about cell lines and Sex and Gender in Research

| | |
|---|---|
| Cell line source(s) | Fibroblast cell line used was the NIH/3T3 line from American Type Culture Collection (ATCC) |
| Authentication | ATCC provides authentication data, cell line history, morphology, and species confirmation. Cell lines were confirmed to be myocplasma free and were observed to be of the expected morphology. |
| Mycoplasma contamination | Cell lines were negative for mycoplasma |
| Commonly misidentified lines (See ICLAC register) | N/A |

## Animals and other research organisms

Policy information about studies involving animals; ARRIVE guidelines recommended for reporting animal research, and Sex and Gender in Research

| | |
|---|---|
| Laboratory animals | C57BL/6 mice of 8-10 weeks age were infected with a low dose (~20 eggs) of Trichuris muris Edinburgh (E) strain by oral gavage |
| Wild animals | No wild animals were used in this study |
| Reporting on sex | Sex-based analysis was not performed in this study since the fundamental mechanisms governing susceptibility and resistance to Trichuris muris infection are known to remain consistent across sexes |
| Field-collected samples | Samples were not collected from the field |
| Ethics oversight | All procedures were performed in accordance with the United Kingdom's Animals (Scientific Procedures) act (1986) under Home Office project license P043A3082 and after local AWERB approval |

Note that full information on the approval of the study protocol must also be provided in the manuscript.

## Plants

| | |
|---|---|
| Seed stocks | *Report on the source of all seed stocks or other plant material used. If applicable, state the seed stock centre and catalogue number. If plant specimens were collected from the field, describe the collection location, date and sampling procedures.* |
| Novel plant genotypes | *Describe the methods by which all novel plant genotypes were produced. This includes those generated by transgenic approaches, gene editing, chemical/radiation-based mutagenesis and hybridization. For transgenic lines, describe the transformation method, the number of independent lines analyzed and the generation upon which experiments were performed. For gene-edited lines, describe the editor used, the endogenous sequence targeted for editing, the targeting guide RNA sequence (if applicable) and how the editor was applied.* |
| Authentication | *Describe any authentication procedures for each seed stock used or novel genotype generated. Describe any experiments used to assess the effect of a mutation and, where applicable, how potential secondary effects (e.g. second site T-DNA insertions, mosiacism, off-target gene editing) were examined.* |
